# Supplementary material for: Electron-Induced Repair of 2′-Deoxyribose Sugar Radicals in DNA: A Density Functional Theory (DFT) Study
Source: Int J Mol Sci. 2021 Feb 9;22(4):1736. doi: 10.3390/ijms22041736 (PMC7916153; doi:10.3390/ijms22041736)
Supplement: Supplementary file 1 [file ijms-22-01736-s001.pdf]

Supporting Information

Electron-Induced Repair of 2'-Deoxyribose Sugar Radicals in DNA: A Density Functional Theory (DFT) Study

Michael Bell, Anil Kumar and Michael D. Sevilla
Department of Chemistry, Oakland University, Rochester, MI 48309, USA

Table of contents

1. Figure S1- ωB97XD-PCM/6-31++G** calculated Mulliken spin density distribution of sugar radicals ($C_{1'}^{•}$, $C_{2'}^{•}$, $C_{3'}^{•}$, $C_{4'}^{•}$ and $C_{5'}^{•}$) of 2'-dT (left column) and HOMO of one-electron reduced corresponding sugar radicals (anions) of 2'-dT (right column). Spin density calculated at 0.004 electron/bohr^3 and HOMO is calculated at 0.02 electron/bohr^3, respectively.

2. Figure S2- ωB97XD-PCM/6-31++G** calculated optimized structures of (i) 5'(R),8-Cyclo-2'-deoxyguanosine (left) and (ii) 5'(S),8-Cyclo-2'-deoxyguanosine (right) in their neutral state (diamagnetic).

3. Figure S3- ωB97XD-PCM/6-31++G** calculated HOMO plots of sugar anions ($C_{2'}^{-}$, $C_{3'}^{-}$, $C_{4'}^{-}$, and $C_{5'}^{-}$) of 2'-dT + $3H_2O$ after proton transfer. These sugar anions are protonated from solvent without any substantial barrier and repaired to normal 2'-dT leaving OH^- + $2H_2O$.

4. Table S1- The ωB97XD-PCM/6-31++G** calculated relative free energies (ΔG), relative populations based on Boltzmann distribution of (i) 5'(R),8-Cyclo-2'-deoxyguanosine and (ii) 5'(S),8-Cyclo-2'-deoxyguanosine in their neutral state (diamagnetic).

5. ωB97XD-PCM/6-31++G** calculated SCF and free energies (G) in A.U. of species considered in this work.

6. Two Methods of Calculation of E^o for reactions involving protonation.

7. Table S2- The ωB97XD-PCM/6-31++G** calculated one-electron reduction potential (E^o) of sugar radicals of 2'-dG using two methods:

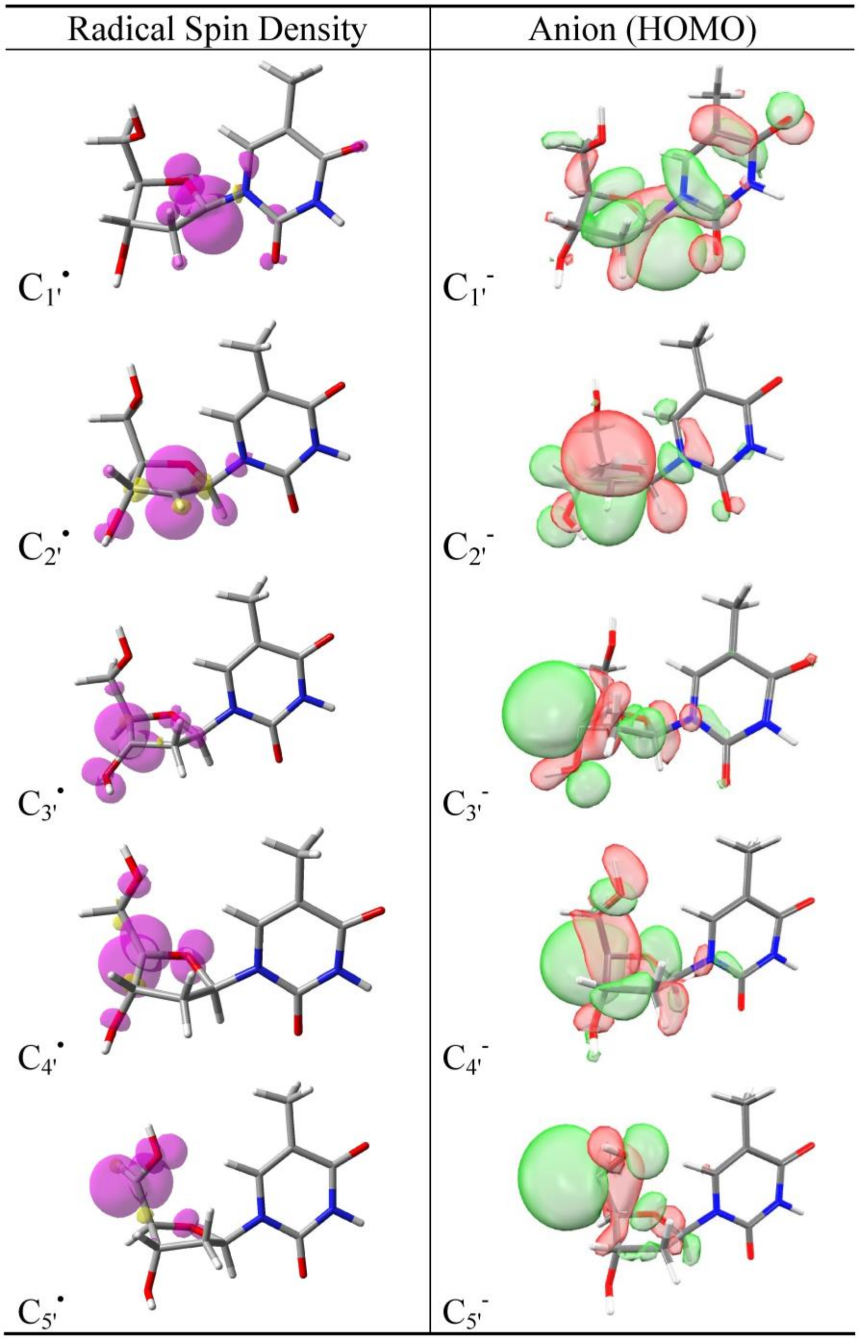

Figure S1- ωB97XD-PCM/6-31++G** calculated Mulliken spin density distribution of sugar radicals ($C_{1'}^{\bullet}$, $C_{2'}^{\bullet}$, $C_{3'}^{\bullet}$, $C_{4'}^{\bullet}$ and $C_{5'}^{\bullet}$) of 2'-dT (left column) and HOMO of one-electron reduced corresponding sugar radicals (anions) of 2'-dT (right column). Spin density calculated at 0.004 electron/bohr^3 and HOMO is calculated at 0.02 electron/bohr^3, respectively.

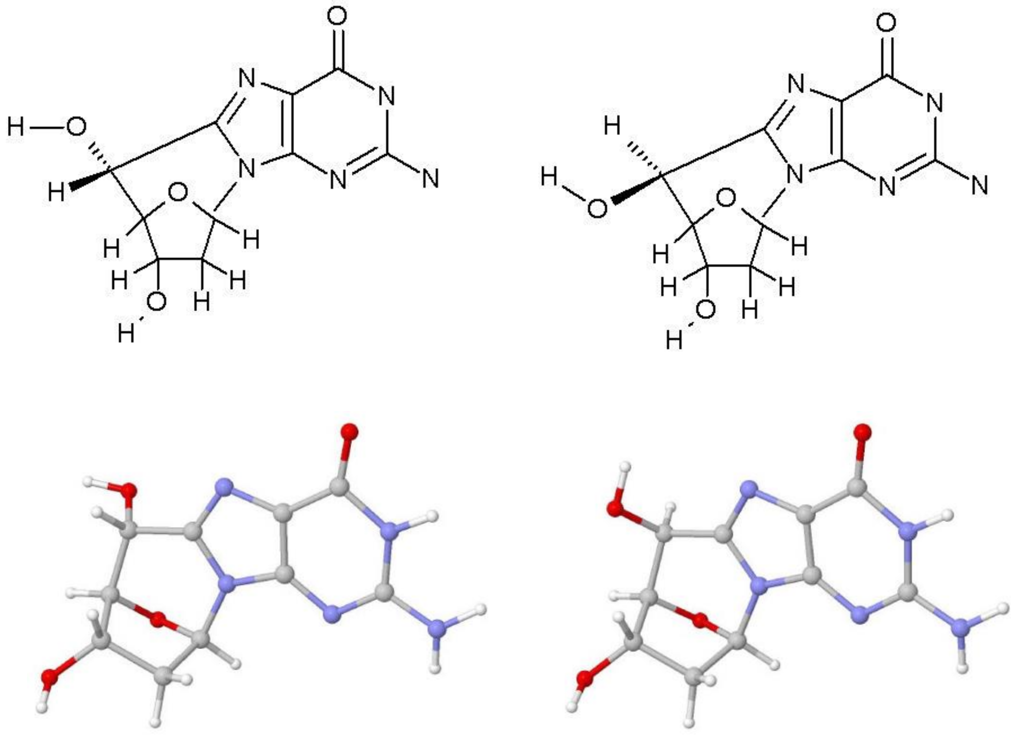

5'(R),8-Cyclo-2'-deoxyguanosine 5'(S),8-Cyclo-2'-deoxyguanosine

Figure S2- ωB97XD-PCM/6-31++G** calculated optimized structures of (i) 5'(R),8-Cyclo-2'-deoxyguanosine (left) and (ii) 5'(S),8-Cyclo-2'-deoxyguanosine (right) in their neutral state (diamagnetic).

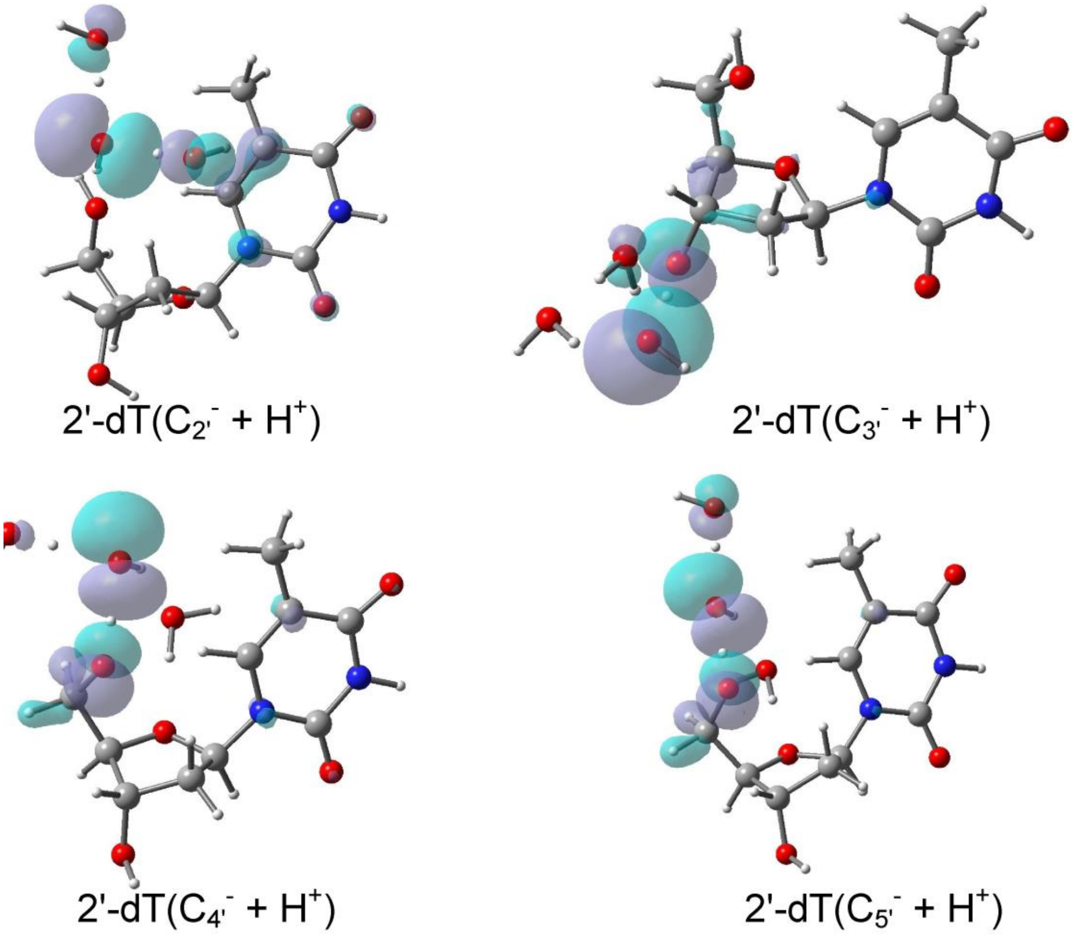

Figure S3- ωB97XD-PCM/6-31++G** calculated HOMO plots of sugar anions ($C_{2'}^{-}$, $C_{3'}^{-}$, $C_{4'}^{-}$, and $C_{5'}^{-}$) of 2'-dT + $3H_2O$ after proton transfer. These sugar anions are protonated from solvent without any substantial barrier and repaired to normal 2'-dT leaving OH^- + $2H_2O$.

Table S1- The ωB97XD-PCM/6-31++G** calculated relative free energies (ΔG), relative populations based on Boltzmann distribution of (i) 5'(R),8-Cyclo-2'-deoxyguanosine and (ii) 5'(S),8-Cyclo-2'-deoxyguanosine in their neutral state (diamagnetic).

5',8-cyclo-2'-dG<br>ωB97XD-PCM/6-31++G**			
Species	ΔG (kJ/mol)	Population (%) at 298 K	Free energy (G) in A.U.
5'(R)[a]	2.29	28.4	-961.963008
5'(S)[b]	0.00	71.6	-961.963880

[a]5'(R),8-Cyclo-2'-deoxyguanosine, see Figure S3.
[b]5'(S),8-Cyclo-2'-deoxyguanosine, see Figure S3.

5. ωB97XD-PCM/6-31++G** calculated electronic (TE) and free energies (G) in A.U. of species considered in this work.

**2'-deoxyguanosine (2'-dG)**			
Species	TE (A.U.)	Free Energy (G) (A.U.)	
Neutral 2'-dG	-963.360873055	-963.150565	
C1 radical	-962.699944307	-962.505228	
C2 radical	-962.687884564	-962.494536	
C3 radical	-962.695404865	-962.499903	
C4 radical	-962.697856995	-962.505565	
C5 radical	-962.697603039	-962.502819	
**Anion (radical + e-)**			
C1 anion	-962.797559321	-962.602105	
C2 anion	-962.808828767	-962.614542	
C3 anion	-962.796414394	-962.601507	
C4 anion	-962.800744761	-962.605668	
C5 anion	-962.793320512	-962.598932	

**2'-deoxythymidine (2'-dT)**			
Species	TE (A.U.)	Free Energy (G) (A.U.)	
Neutral 2'-dT	-874.956492875	-874.747206	
C1 radical	-874.293580345	-874.100197	
C2 radical	-874.283361199	-874.091767	
C3 radical	-874.291363270	-874.097579	
C4 radical	-874.292279361	-874.100751	
C5 radical	-874.293503302	-874.098981	
**Anion (radical + e-)**			

C1 anion	-874.391317264	-874.197678	
C2 anion	-874.400691662	-874.209602	
C3 anion	-874.392130334	-874.198671	
C4 anion	-874.396867980	-874.203537	
C5 anion	-874.394181003	-874.200915	

**5',8-cyclo-2'-Deoxyguanosine**			
Species	TE (A.U.)	Free Energy (G) (A.U.)	
5'(R),8-cyclo-2'-Deoxyguanosine-7-yl radical	-962.712374251	-962.512884	
5'(S),8-cyclo-2'-Deoxyguanosine-7-yl radical	-962.713096661	-962.512336	
**Anion (radical + e-)**			
5'(R),8-cyclo-2'-Deoxyguanosine-7-yl	-962.816291824	-962.617565	
5'(S),8-cyclo-2'-Deoxyguanosine-7-yl	-962.826694097	-962.626692	

6. Two Methods of Calculation of E^o for reactions involving protonation.

In the main text we computed the E^o for the process of reduction for the half reaction:

$$C_{n'\cdot} + e^- + H^+ \rightarrow C_{n'}H \qquad (n = 1\text{-}5) \qquad (3)$$

vs the SHE which is given by the reaction:

$$H^+ + e^- \rightarrow {}^1\!/_2 H_2 \qquad (4)$$

Since reaction (3) minus that of reaction (4) gives reaction 5:

$$C_{n'\cdot} + {}^1\!/_2 H_2 \rightarrow C_{n'}H \qquad (5)$$

The free energy change found for reaction (5) gives the potential for reaction (3) vs the SHE, i.e.,$E^o = -\Delta G^o(5)/RT$.

The usual method is to calculate the energy changes for reaction 3 and combine that with the IUPAC value of the SHE of 4.44 eV (reaction 4). This method requires the solvation energy of the proton which is reported to be ca. -266 kcal/mol or -11.53 eV

(Thapa, B., Schlegel, H. B., J. Phys. Chem. A 2015, 119, 5134−5144. doi.org/10.1021/jp5088866) |

We find that both methods give results within 0.08 eV but the method utlilizing reaction 5 only eliminates the uncertainty in the solvation energy of the proton and the variation in reported values for the SHE.

Table S2- The ωB97XD-PCM/6-31++G** calculated one-electron reduction potential (E^o) of sugar radicals of 2'-dG using two methods: Method 1 given in main text is based on eq. 5. Method 2. Employs the SHE and solvation energy of the proton.

	2'-Deoxyguanosine[a] ωB97XD-PCM/6-31++G**		
	E^o *vs* SHE	(Volt)	
	Method 1[a]	Method 2[b]	
$C_{1'}$	-1.50	-1.58	
$C_{2'}$	-1.79	-1.88	
$C_{3'}$	-1.65	-1.73	
$C_{4'}$	-1.49	-1.58	
$C_{5'}$	-1.57	-1.65	
			Average dev. 0.08V

[a] Method 1: E^o of protonated anion (see eq 5). Value of 1/2(H2) in eq. (5) was calculated using G4 level of theory.
[b.] Method 2 Employs the SHE potential of 4.44 V (equation 4) and solvation energy of the proton as 11.53 eV (in equation 3)..
